# Interaction of direct and social genetic effects with feeding regime in growing rabbits

**DOI:** 10.1186/s12711-017-0333-2

**Published:** 2017-07-20

**Authors:** Miriam Piles, Ingrid David, Josep Ramon, Laurianne Canario, Oriol Rafel, Mariam Pascual, Mohamed Ragab, Juan P. Sánchez

**Affiliations:** 1 Institute for Food and Agriculture Research and Technology, Torre Marimon s/n, 08140 Caldes de Montbui, Barcelona Spain; 2 GenPhySE, INRA, Université de Toulouse, INPT, ENVT, 31326 Castanet Tolosan, France; 3 Poultry Production Department, Kafr El-Sheikh University, Kafr El-Sheikh, 33516 Egypt

## Abstract

**Background:**

Most rabbit production farms apply feed restriction at fattening because of its protective effect against digestive diseases that affect growing rabbits. However, it leads to competitive behaviour between cage mates, which is not observed when animals are fed ad libitum. Our aim was to estimate the contribution of direct ($d$) and social ($s$) genetic effects (also known as indirect genetic effects) to total heritable variance of average daily gain (${\text{ADG}}$) in rabbits on different feeding regimens (FR), and the magnitude of the interaction between genotype and FR (G × FR).

**Methods:**

A total of 6264 contemporary kits were housed in cages of eight individuals and raised on full ($F$) or restricted ($R$) feeding to 75% of the ad libitum intake. A Bayesian analysis of weekly records of ${\text{ADG}}$ (from 32 to 60 days of age) in rabbits on $F$ and $R$ was performed with a two-trait model including $d$ and $s$.

**Results:**

The ratio between total heritable variance and phenotypic variance ($T^{2}$) was low (<0.10) and did not differ significantly between FR. However, the ratio between $h^{2}$ (i.e. variance of $d$ relative to phenotypic variance) and $T^{2}$ was ~0.52 and 0.86 for animals on $R$ and $F$, respectively, thus $s$ contributed more to the heritable variance of animals on $R$ than on $F$. Feeding regimen also affected the sign and magnitude of the correlation between $d$ and $s$, i.e. −0.5 and ~0 for animals on $R$ and $F$, respectively. The posterior mean (posterior sd) of the correlation between estimated total breeding values (ETBV) of animals on $R$ and $F$ was 0.26 (0.20), indicating very strong G × FR interactions. The correlations between $d$ and $s$ in rabbits on $F$ and $R$ ranged from −0.47 ($d$ on $F$ and $s$ on $R$) to 0.64.

**Conclusions:**

Our results suggest that selection of rabbits for ${\text{ADG}}$ under $F$ may completely fail to improve ${\text{ADG}}$ in rabbits on $R$. Social genetic effects contribute substantially to ETBV of rabbits on $R$ but not on $F$. Selection for ${\text{ADG}}$ should be performed under production conditions regarding the FR, by accounting for $s$ if the amount of food is limited.

## Background

Feed efficiency is a key factor of profitability, productivity and sustainability of rabbit meat production. However, direct selection for this trait is difficult to implement because it requires individual recording of feed intake (${\text{FI}}$), which is expensive and time consuming for animals housed in individual cages, and not possible for animals housed in groups since automatic feeding systems are still not available for this species. As a consequence, selection for feed efficiency has been performed either by indirect selection for average daily gain (${\text{ADG}}$) of animals fed ad libitum and housed in groups [1], or by direct selection for residual feed intake (${\text{RFI}}$) or for ${\text{ADG}}$ under restricted feeding [2] with a limited number of selection candidates kept in individual cages. Results that compare the production performance of young rabbits selected for ${\text{ADG}}$ and ${\text{RFI}}$ and bred under different feeding regimens (FR) suggest there is an interaction effect between genotype and FR (G × FR) on ${\text{ADG}}$ but not on other traits such as body weight (${\text{BW}}$), ${\text{FI}}$ or feed efficiency [2]. However, to date, variance estimates due to the G × FR interaction or its components (i.e. difference in genetic variances and genetic correlation between different conditions) for production traits in rabbit have not been reported. This interaction effect could be relevant when animals are bred in collective cages, which is the most common practice on commercial rabbit farms and elicits competition for feed intake between cage mates.

Social effects might be particularly important when feed restriction is applied at fattening, which is a common practice on production farms to reduce mortality associated to digestive disorders that are caused by some diseases, such as epizootic rabbit enteropathy [3]. By restricting the amount of food to 75% of the ad libitum intake and providing it once a day, Dalmau et al. [4] observed that signs of antagonistic behaviour such as biting, displacement and animals jumping one on top of each other occurred during the whole growing period. However, they did not find any effect of feed restriction on the coefficient of variation in body weight, which could indicate that, in spite of the competition, all kits faced the same level of feed restriction [5].

The benefits of selection for feed efficiency in individual cages could be lost when animals are kept in collective cages if substantial G × FR interaction effects exist. In the presence of those effects, phenotypic differences among individuals are not the same under different management conditions, with possible re-ranking of individuals [6]. In addition, ignoring the existence of social interaction effects in a breeding program could have negative consequences on the magnitude and sign of response to selection, which depend on the genetic parameters for direct and social genetic effects. Selection for individual performance may lead to strong competition when the covariance between direct and social effects is negative. Then, response to selection, which is determined by the sign of the covariance between an individual’s phenotypic trait value and its total breeding value [7], could take the opposite direction to that desired [8, 9].

The current study aimed at estimating the genetic parameters for direct and social effects on ${\text{ADG}}$ of growing rabbits that were raised on an ad libitum or restricted FR, and the interaction effect between the individual genotype [i.e. total breeding value (${\text{TBV}}$)] and FR.

## Methods

### Animals and housing conditions

The experiment was carried out between July 2012 and June 2014 on the experimental farm of IRTA in Spain. We used 7864 kits, which were produced from a rabbit sire line (Caldes line [10]) selected for ${\text{ADG}}$ in kits fed ad libitum during the fattening period (from 32 to 60 days of age) and housed in cages of eight individuals on a nucleus farm. All animals in the experiment were bred under the same management conditions except for the FR at fattening (5 weeks long in this experiment), which was either (1) full feeding i.e. ad libitum ($F$) or (2) restricted feeding ($R$) to 75% of the ad libitum feed intake, with in both cases, the same standard diet. After weaning at 32 days of age, kits were randomly assigned to one of these two FR. In order to obtain homogeneous groups regarding animal size, kits under a FR were assigned to two groups based on their ${\text{BW}}$: big size kits (BS, i.e. with a ${\text{BW}}$ >700 g) and small size kits (SS, i.e. with a ${\text{BW}}$ ≤700 g), which is a common management practice on rabbit farms to obtain homogeneous growth and body weight at slaughter. Animals from a same litter were distributed to both FR. A maximum of two kits per litter were allocated to the same cage in order to minimize the effect of maternal and pre-weaning environmental effects on behaviour and growth performance at fattening. Kits were housed on a farm close to the selection nucleus (6.2 km) in 969 cages, each containing eight rabbits. Cages assigned to each group were interleaved on the farm.

The fattening period of the experiment lasted 5 weeks and food was supplied once per day in a feeder with three places and in the form of commercial pellets for rabbits that contain antibiotics to control gut disorders. At the last week of fattening, it was changed to a standard food without drugs. Data from this period were not considered for analysis due to the possible impact of the change in diet on the results. Details on the composition of the food for the analysed period are in Table 1. Water was available ad libitum (one nipple drinker per cage). The surface of the cage was 0.38 m^2^. All these housing conditions are considered as standard conditions on commercial farms.

**Table 1:** Feed composition on a wet basis

Component	Amount
Crude fibre (%)	18.70
Crude protein (%)	15.02
Ashes (%)	8.97
Ether extract (%)	3.28
Oxytetracycline (ppm)	400
Valnemulin (ppm)	30
Colistin (ppm)	100

To obtain a feed restriction of 75% of the ad libitum feed intake, the amount of food supplied during week $i$ was computed as 0.75 times the average feed intake of kits on $F$ in a specific group $j$ ($j$ = BS or SS) during the week before (i.e., $i - 1$), plus 10% corresponding to the estimated increase in ${\text{FI}}$ as the animals grows, i.e.:

$${\text{FI}}_{R,ji} = \left( {0.75 + 0.10} \right) \times {\text{FI}}_{F,j(i - 1)} \quad {\text{for}}\;i = 1,2,3,4\;{\text{and}}\;5\;{\text{and}}\;j = {\text{BS}}\;{\text{or}}\;{\text{SS}}.$$

This amount of food was multiplied by the number of animals alive in each cage at that time to determine feed requirements of the group. The amount of food for week 1 was computed from data that were recorded in previous experiments on the same line with animals raised in the same season. Actual feed restriction was on average 75 and 74.1% of the ad libitum intake in BS and SS kits, respectively.

Individual ${\text{BW}}$ and total ${\text{FI}}$ of kits in the same cage were weekly recorded after weaning (32 days). Average daily gain for a specific week was calculated as the difference in ${\text{BW}}$ at the beginning and end of the week divided by the number of days elapsed (i.e., 7 ± 1 day). On control days, food was supplied after the kits were weighed. Information on sick animals was recorded. For each week, groups with animals that presented symptoms of an infectious disease, which was not caused a priori by antagonist behaviour (e.g. epizootic rabbit enteropathy, ERE, or respiratory problems), were discarded from the analyses, so that group size was always equal to 8. The average number of weeks with records per cage was 3.45 for animals on $F$ and 3.41 for animals on $R$. The distribution of the data for each FR, ${\text{BW}}$ class and week, after data filtering, is in Table 2. The final set of data for analysis included information on 6264 individuals born from 1303 litters housed in 783 cages. The pedigree included information on 7701 individuals, tracing back 5 generations from that corresponding to the animals of the experiment. The average relatedness coefficient within a cage was equal to 0.16.

**Table 2:** Distribution of the number of records

Feeding regimen	Class according to body weight at weaning	Week of fattening			
		1	2	3	4
Restricted	Small	124	108	97	76
	Big	212	220	188	167
Full	Small	122	109	83	78
	Big	258	233	182	161

### Models and statistical analyses

Preliminary analyses of the data were performed using mixed linear models that were implemented with “lme4” and “lsmeans” packages of the R software [11]. Weekly records of ${\text{BW}}$, ${\text{ADG}}$ and their coefficient of variation (${\text{CV}}$) within a cage were analysed. Analysis of ${\text{BW}}$ and ${\text{ADG}}$ included the random effects of animal, cage and litter and the systematic effects of FR (two levels: $F$ and $R$), week of fattening (four levels), body weight at weaning (two levels: BS and SS), batch (14 levels), parity order (four levels: 1, 2, 3, >3), number of kits born alive in the litter (seven levels: <6, 6, 7, 8, 9, 10, >10) and the interaction between week and all other systematic effects in the final model. Triple interactions between all systematic effects were initially included but finally discarded because they were not significant. Models for the analysis of weekly ${\text{CV}}$ in ${\text{BW}}$ and ${\text{ADG}}$ included the random effect of cage and the systematic effects of week of fattening, FR, ${\text{BW}}$ at weaning, batch and the interactions between week and other systematic effects.

The genetic analysis was performed in two steps. In a first step, in order to estimate the G × FR interaction, ${\text{ADG}}$ of animals on $F$ and $R$ (${\text{ADG}}_{F}$ and ${\text{ADG}}_{R}$, respectively) were considered as different but correlated traits and analysed with a two-trait model. The following repeatability animal model was fitted to weekly records of the same trait:

Model 1 $${\mathbf{y}} = {\mathbf{X}}{\varvec{\upbeta}} + {\mathbf{Z}}_{\text{D}} {\mathbf{d}} + {\mathbf{Z}}_{\text{P}} {\mathbf{p}} + {\mathbf{Z}}_{\text{L}} {\mathbf{l}} + {\mathbf{Z}}_{\text{G}} {\mathbf{g}} + {\mathbf{e}},$$

where ${\mathbf{y}}$ is the vector of ${\text{ADG}}_{F}$ or ${\text{ADG}}_{R}$, ${\varvec{\upbeta}}$ is the vector of systematic effects with the corresponding incidence matrix ${\mathbf{X}}$, ${\mathbf{d}}$ is the vector of additive direct genetic effects with the corresponding incidence matrix ${\mathbf{Z}}_{\text{D}}$, ${\mathbf{p}}$ is the vector of permanent animal effects (6264 levels) with the corresponding incidence matrix ${\mathbf{Z}}_{\text{P}}$, ${\mathbf{l}}$ is the vector of litter birth effects (1303 levels) with the corresponding incidence matrix ${\mathbf{Z}}_{\text{L}}$, ${\mathbf{g}}$ is the vector of non-genetic group effects (783 levels) with the corresponding incidence matrix ${\mathbf{Z}}_{\text{G}}$, and ${\mathbf{e}}$ is the vector of residuals. Systematic effects were the same as those that were finally included in the preliminary analysis of ${\text{ADG}}$ except FR and its interaction with week of fattening.

In a second step, the previous model was extended to include social genetic effects between cage mates [12]. This model can be written as:

Model 2 $${\mathbf{y}} = {\mathbf{X}}{\varvec{\upbeta}} + {\mathbf{Z}}_{\text{D}} {\mathbf{d}} + {\mathbf{Z}}_{\text{S}} {\mathbf{s}} + {\mathbf{Z}}_{\text{P}} {\mathbf{p}} + {\mathbf{Z}}_{\text{L}} {\mathbf{l}} + {\mathbf{Z}}_{\text{G}} {\mathbf{g}} + {\mathbf{e}},$$

where ${\mathbf{d}}$ and ${\mathbf{s}}$ are the vectors of the direct and social genetic effects, respectively and ${\mathbf{Z}}_{\text{D}}$ and ${\mathbf{Z}}_{\text{S}}$ are their corresponding incidence matrices. All other terms are identical to Model 1.

Bayesian methodology was used to estimate model parameters. The prior distribution of the additive genetic values was ${\mathbf{d}}|{\mathbf{G}}_{1} \,\sim\,N\left( {{\mathbf{0}},{\mathbf{A}} \otimes {\mathbf{G}}_{1} } \right)$ for Model 1, where ${\mathbf{A}}$ is the matrix of coefficients of relatedness between individuals, $\otimes$ denotes the Kronecker product and ${\mathbf{G}}_{1}$ is the 2 × 2 additive genetic covariance matrix for ${\text{ADG}}$ of animals on $F$ and $R$:

$${\mathbf{G}}_{1} = \left[ {\begin{array}{*{20}c} {\sigma_{dF}^{2} } & {\quad \sigma_{dF,dR} } \\ {\sigma_{dR,dF} } & {\quad \sigma_{dR}^{2} } \\ \end{array} } \right].$$

For Model 2, the prior distribution of the additive genetic values was $$\left. {\left[ {\begin{array}{*{20}c} {\mathbf{d}} \\ {\mathbf{s}} \\ \end{array} } \right]} \right|{\mathbf{G}}_{2} {\mkern 1mu} \sim{\mkern 1mu} N\left( {{\mathbf{0}}, {\mathbf{G}}_{2} \otimes {\mathbf{A}}} \right)$$, where all the terms are defined as before except ${\mathbf{G}}_{2}$ which, in this case, is the 4 × 4 additive genetic covariance matrix of direct and social effects for ${\text{ADG}}$ of animals on $F$ and $R$:

$${\mathbf{G}}_{2} = \left[ {\begin{array}{*{20}c} {\sigma_{dF}^{2} } & {\quad \sigma_{dF,dR} } & {\quad \sigma_{dF,sF} } & {\quad \sigma_{dF,sR} } \\ {\sigma_{dR,dF} } & {\quad \sigma_{dR}^{2} } & {\quad \sigma_{dR,sF} } & {\quad \sigma_{dR,sR} } \\ {\sigma_{sF,dF} } & {\quad \sigma_{sF,dR} } & {\quad \sigma_{sF}^{2} } & {\quad \sigma_{sF,sR} } \\ {\sigma_{sR,dF} } & {\quad \sigma_{sR,dR} } & {\quad \sigma_{sR,sF} } & {\quad \sigma_{sR}^{2} } \\ \end{array} } \right],$$

where, $\sigma_{ij}^{2}$ is the variance of direct ($i = d$) or social ($i = s$) additive genetic effects for ${\text{ADG}}$ of animals on full ($j = F$) or restricted ($j = R$) feeding; and $\sigma_{{ij,i^{\prime } j^{\prime } }}$ is the covariance terms for $ij \ne i^{\prime } j^{\prime }$ with ($i$ and $i^{\prime }$ = $d$ or $s$) and ($j$ and $j^{\prime }$ = $F$ or $R$).

The prior distribution of the litter effects $\left( {l_{i} , i = 1, \ldots ,{\text{N}}_{\text{l}} } \right)$, group effects $\left( {g_{i} , i = 1, \ldots ,{\text{N}}_{g} } \right)$ and permanent effects $\left( {p_{i} , i = 1, \ldots ,{\text{N}}_{p} } \right)$ in both models were ${\mathbf{l}}|{\mathbf{L}}\,\sim\,N\left( {{\mathbf{0}}, {\mathbf{I}} \otimes {\mathbf{L}}} \right)$, ${\mathbf{g}}|{\mathbf{G}}\,\sim\,N\left( {{\mathbf{0}}, {\mathbf{I}} \otimes {\mathbf{Gr}}} \right)$, and ${\mathbf{p}}|{\mathbf{p}}\,\sim\,N\left( {{\mathbf{0}},{\mathbf{I}} \otimes {\mathbf{P}}} \right)$, respectively, where ${\mathbf{l}}$, ${\mathbf{g}}$ and ${\mathbf{p}}$ are the corresponding vectors of litter, group and permanent effects, respectively, and ${\mathbf{L}}$, ${\mathbf{Gr}}$ and ${\mathbf{P}}$ are the corresponding 2 × 2 covariance matrices defined as: $${\mathbf{L}} = \left[ {\begin{array}{*{20}c} {\sigma_{lF}^{2} } & {\quad \sigma_{lF,lR} } \\ {\sigma_{lR,lF} } & {\quad \sigma_{lR}^{2} } \\ \end{array} } \right]$$, $${\mathbf{Gr}} = \left[ {\begin{array}{*{20}c} {\sigma_{gF}^{2} } & {\quad 0} \\ 0 & {\quad \sigma_{gR}^{2} } \\ \end{array} } \right]$$ and $${\mathbf{P}} = \left[ {\begin{array}{*{20}c} {\sigma_{pF}^{2} } & {\quad 0} \\ 0 & {\quad \sigma_{pR}^{2} } \\ \end{array} } \right]$$, where $\sigma_{ij}^{2}$ is the variance of litter ($i = l$) or group effects ($i = g$) of animals on $F$ or $R$ ($j = F\;{\text{or}}\;R$) and $\sigma_{lF,lR}$ is the covariance between litter effects of animals on $F$ and $R$. ${\mathbf{I}}$ is the identity matrix. ${\text{N}}_{l}$, ${\text{N}}_{g}$ and ${\text{N}}_{p}$ are the number of litters, groups and animals with records, respectively. The residual variance matrix was defined as $${\mathbf{R}} = \left[ {\begin{array}{*{20}c} {\sigma_{eF}^{2} } & {\quad 0} \\ 0 & {\quad \sigma_{eR}^{2} } \\ \end{array} } \right]$$, where $\sigma_{eF}^{2}$ and $\sigma_{eR}^{2}$ are the residual variances for animals on $F$ and $R$, respectively.

Flat priors were used for systematic effects and variance components of the animal mixed models. The marginal posterior distributions of all the unknowns were approximated by Gibbs sampling [13] using the gibbs2f90 software [14].

Two sampling processes of 1,500,000 iterations each were run. The first 100,000 iterations were discarded as burn-in. One sample of the parameters of interest was saved every 100 iterations. The sampling variance of the chains was obtained by computing Monte Carlo standard errors [15]. Statistics for the marginal posterior distributions were calculated directly from the samples.

Ratios of the phenotypic variance under FR $j$ ($j = F\;{\text{or}}\;R$), $\sigma_{Pj}^{2}$, were computed from the variance components of the different models. In all models, the contribution of the additive genetic variance to the total phenotypic variance for each trait was computed as [16]:

1 $$\sigma_{dj}^{2} + \left( {n - 1} \right)\sigma_{sj}^{2} + r_{j} \times \left( {n - 1} \right) \times \left[ {2 \sigma_{dj,sj} + \left( {n - 2} \right)\sigma_{sj}^{2} } \right],$$

where $\sigma_{dj}^{2}$ and $\sigma_{sj}^{2}$ are the variances of direct and social additive genetic effects, respectively; $\sigma_{dj,sj}$ is the covariance between $\sigma_{dj}^{2}$ and $\sigma_{sj}^{2}$; $n$ is the number of cage mates (8 in our case); and $r_{j}$ is the average relationship coefficient between all pairs of individuals within a cage.

The total heritable contribution of the genes of a single individual on the mean trait value, defined as the individual’s total breeding value (TBV) [17], was computed for individual $i$ under FR $j$ as:

2 $${\text{TBV}}_{ij} = d_{ij} + \left( {n - 1} \right)s_{ij} ,$$

the total heritable variance available for selection $\sigma_{TBV}^{2}$ [18] was calculated as follows:

3 $$\sigma_{{TBV_{j} }}^{2} = \sigma_{dj}^{2} + 2\left( {n - 1} \right)\sigma_{dj,sj} + \left( {n - 1} \right)^{2} \sigma_{sj}^{2} ,$$

and the ratio between total heritable variance and phenotypic variance [19] was calculated as follows:

4 $$T_{j}^{2} = \frac{{\sigma_{{{\text{TBV}}_{j} }}^{2} }}{{\sigma_{{P_{j} }}^{2} }}.$$

The covariance of ${\text{TBV}}$ between FR was computed as:

5 $$Cov\left( {{\text{TBV}}_{F} , {\text{TBV}}_{R} } \right) = \sigma_{dF,dR} + \left( {n - 1} \right)\sigma_{dF,sR} + \left( {n - 1} \right)\sigma_{sF,dR} + \left( {n - 1} \right)^{2} \sigma_{sF,sR} ,$$

where $\sigma_{dF,dR}$ is the covariance between direct genetic effects for animals on $F$ and $R$, $\sigma_{dF,sR}$ is the covariance between direct genetic effects of animals on $F$ and social genetic effects of animals on $R$, $\sigma_{sF,dR}$ is the covariance between social genetic effects of animals on $F$ and direct genetic effects of animals on $R$, $\sigma_{sF,sR}$ is the covariance between social genetic effects of animals on $F$ and $R$, and $n$ is the number of kits in a cage (8 in our analysis).

The deviance information criterion (DIC [20]) was used to compare the models and assess which one yielded the best fit (considering a penalty for model complexity) to the data.

## Results

### Phenotypic analysis of BW and ADG

In this experiment, we confirmed that feed restriction has a protective effect on the health of growing rabbits with the mortality rate being 14.6% for animals on $F$ and 9.5% for animals on $R$. The FR also had an important effect on ${\text{BW}}$ and ${\text{ADG}}$, as expected, with slightly different values between large and small kits at weaning. Thus, the overall means for ${\text{BW}}$ were equal to 1773 and 1487 g for BS kits on $F$ and $R$, respectively, and 1460 and 1164 g for SS kits on $F$ and $R$, respectively. The overall means for ${\text{ADG}}$ were equal to 51.9 and 36.9 g/day for BS kits on $F$ and $R$, respectively, and 49.6 and 34.4 g/day for SS kits on $F$ and $R$, respectively.

**Fig. 1:**
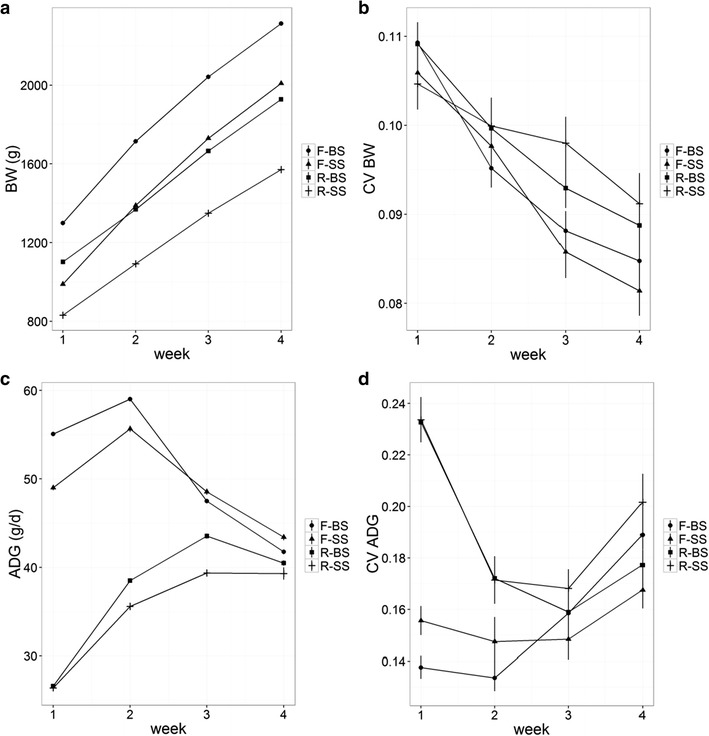
Mean (**a**, **c**) and coefficient of variation within cage (${\text{CV}}$; **b**, **d**) in body weight at the end of each week (BW) and average daily gain (ADG) for growing rabbits of big (BS) or small (SS) weaning body weight on full ($F$) or restricted ($R$) feeding regimen during the fattening period

The pattern of growth also differed between animals on $F$ or $R$ (Fig. 1). Post-weaning growth decelerated after the first week for animals on $F$ whereas it accelerated until week 3 and then remained constant for animals on $R$ (Fig. 1c). The mean ${\text{ADG}}$ was 106.9% higher for animals on $F$ than on $R$ for week 1 [difference in LSmeans ± standard error (se) = 26.8 ± 0.3 g/day] but it decreased to 4.5% for week 4 (difference in LSmeans ± se = 1.8 ± 0.4 g/day).

Variation in growth rate between cage mates was larger for animals on $R$ than on $F$ for the first 2 weeks after weaning. The magnitude of the difference was 0.091 ± 0.006 and 0.034 ± 0.006 for weeks 1 and 2, respectively (Fig. 1d). On the contrary, no differences between the ${\text{CV}}$ in ${\text{BW}}$ of the four groups of animals were observed for weeks 1 and 2 but small differences were found for weeks 3 and 4, and these were also larger for animals on $R$ than on $F$ (difference = 5.7e−3 ± 2.4e−3 and 5.1e−3 ± 2.5e−3 for weeks 3 and 4, respectively).

### Genetic analysis of ADG when social effects were ignored

The DIC was equal to 231,366.50 for the model that ignored social effects. Ratios of phenotypic variance from the classical repeatability model for ${\text{ADG}}_{F}$ and ${\text{ADG}}_{R}$ are in Table 3. We found a 40% larger phenotypic variance for animals on $F$ than on $R$. This difference was mainly explained by a difference in additive genetic variance which was 4.7 times larger for animals on $F$ than on $R$. Residual variance also differed between FR and was 1.3 times larger for animals on $F$ than on $R$, whereas the variance due to permanent animal effects, litter or group effects did not differ between FR.

**Table 3:** Genetic parameters of average daily gain under different feeding regimens from the classical animal repeatability model

Parameter^a^	Restricted feeding				Full feeding			
	Mean	HPD 95%^b^		MCse^c^	Mean	HPD 95%^b^		MCse^c^
$h^{2}$	0.023	0.009	0.041	0.00047	0.078	0.049	0.108	0.00038
$g^{2}$	0.032	0.020	0.044	0.00007	0.024	0.012	0.036	0.00006
$p^{2}$	0.013	0.001	0.027	0.00029	0.008	0.000	0.021	0.00036
$l^{2}$	0.072	0.053	0.091	0.00026	0.073	0.052	0.096	0.00027
$\sigma_{p}^{2}$	56.014	54.308	57.773	0.00835	78.265	75.813	80.738	0.01194

^a^ $h^{2}$: heritability; $g^{2}$: variance of group effects relative to phenotypic variance; $p^{2}$: variance of permanent animal effects relative to phenotypic variance; $l^{2}$: variance of litter effects relative to phenotypic variance; $\sigma_{p}^{2}$: phenotypic variance

^b^HPD 95%: highest posterior density interval at 95% with lower and upper limits

^c^MCse: Monte Carlo standard error

Differences in variance components between FR are due in part to the reduced magnitude of ${\text{ADG}}$ due to limited feed intake. When the ratios of phenotypic variance were computed for each FR, we found similar values for residual variance (around 0.8), permanent, litter, and group effects but not for additive genetic effects which were quite different. Thus, posterior means (posterior sd) of heritability were 0.08 (0.02) and 0.02 (0.01) for ${\text{ADG}}_{F}$ and ${\text{ADG}}_{R}$, respectively. Posterior means (posterior sd) of additive genetic and litter correlations between FR were 0.81 (0.16) and 0.92 (0.07), respectively (Table 4).

**Table 4:** Correlations between components of average daily gain of growing rabbits between full and restricted feeding regimens from the classical animal model

Effect	Mean	Lower and upper limits of HPD 95%^a^		MCse^b^
Additive	0.807	0.504	1.000	0.01212
Litter	0.924	0.790	1.000	0.00282

^a^HPD 95%: highest posterior density interval at 95%

^b^MCse: Monte Carlo standard error

### Genetic analysis of ADG when social effects were included

**Fig. 2:**
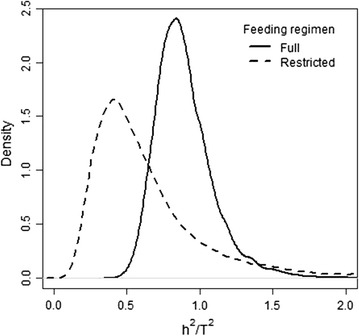
Marginal posterior distribution for the ratio of classical definition of heritability ($h^{2}$) and proportion of total heritable variance relative to phenotypic variance ($T^{2}$) of average daily gain of growing rabbits on full ($F$) and restricted ($R$) feeding regimen (Model 2)

**Fig. 3:**
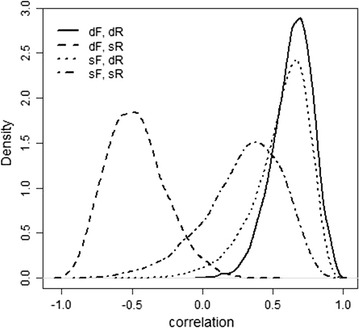
Marginal posterior distribution of the genetic correlations between direct ($d$) and social ($s$) effects for average daily gain of growing rabbits on full ($F$) or restricted ($R$) feeding regimen

The DIC was equal to 231,062.12 for the model that accounted for social effects, which was 304.38 units less than for Model 1. Results of this analysis are in Table 5, Figs. 2 and 3. The marginal posterior distributions of the proportion of phenotypic variance due to group, litter and permanent effects were low and very close for both FR (2, 7 and 1% for group, litter and permanent effects respectively; Table 5). These values did not differ from those obtained when social genetic effects were ignored (Table 3).

**Table 5:** Genetic parameters for average daily gain under different feeding regimens from the model that includes social genetic effects

Parameter^a^	Restricted feeding				Full feeding			
	Mean	HPD 95%^b^		MCse^c^	Mean	HPD 95%^b^		MCse^c^
$TBV$	3.616	0.663	6.981	0.07878	7.484	3.786	11.300	0.08072
$h^{2}$	0.033	0.017	0.051	0.00029	0.082	0.053	0.111	0.00041
$T^{2}$	0.064	0.012	0.123	0.00139	0.095	0.050	0.144	0.00102
$s^{2}$	0.0017	0.0005	0.0030	2.9e−05	0.0003	5.7e−05	0.0006	6.5e−06
$\rho_{d,s}$	−0.505	−0.912	−0.072	0.01059	−0.030	−0.553	0.495	0.01286
$g^{2}$	0.025	0.011	0.038	0.00017	0.023	0.011	0.036	0.00012
$p^{2}$	0.010	0.001	0.021	0.00027	0.008	0.000	0.020	0.00029
$l^{2}$	0.066	0.048	0.084	0.00021	0.071	0.049	0.092	0.00031
$\sigma_{p}^{2}$	56.296	54.590	58.144	0.01309	78.415	75.998	81.045	0.01620

^a^ $TBV$: total breeding value; $h^{2}$: variance of direct genetic effects relative to phenotypic variance; $T^{2}$: variance of $TBV$ relative to phenotypic variance; $s^{2}$: variance of social genetic effects relative to phenotypic variance; $\rho_{d,s}$: correlation between direct and social genetic effects; $g^{2}$: variance of group effects relative to phenotypic variance; $p^{2}$: variance of permanent animal effects relative to phenotypic variance; $l^{2}$: variance of litter effects relative to phenotypic variance; $\sigma_{p}^{2}$: phenotypic variance

^b^HPD 95%: highest posterior density interval at 95%

^c^MCse: Monte Carlo standard error

When estimating social genetic effects, it is essential to account for non-genetic effects between group mates [8]. Variance between groups takes the covariance among individuals within a group into account when it is positive. In a preliminary analysis, we checked that there was a positive correlation between individuals within a group. This correlation was estimated per week and per FR using a model that included social effects but not non-genetic group effects and that considered a uniform correlation between residuals for animals in the same cage. Models were fitted using ASReml [21]. The results obtained showed that the correlation between residuals was positive for all weeks and FR, ranging from 0.07 to 0.20 for ${\text{ADG}}_{F}$ and 0.11 to 0.19 for ${\text{ADG}}_{R}$. Therefore, the covariance between cage mates was estimated from the non-genetic group effects of Model 2.

Estimates of the variance of direct genetic effects did not significantly change when social genetic effects were included in the model (Tables 3, 5). Posterior means (posterior sd) were 6.14 (1.21) and 6.42 (1.20) g/day for ${\text{ADG}}_{F}$ with Models 1 and 2, respectively, and 1.31 (0.50) and 1.86 (0.50) g/day for ${\text{ADG}}_{R}$ with Models 1 and 2, respectively. Variances of social genetic effects were estimated at 0.025 (0.012) and 0.096 (0.038) g/day for ${\text{ADG}}_{F}$ and ${\text{ADG}}_{R}$, respectively.

Heritability (i.e. $h^{2}$, the ratio between variance of direct genetic effects and phenotypic variance) was low (0.08) for ${\text{ADG}}_{F}$ and very low (0.03) for ${\text{ADG}}_{R}$. The ratio between total heritable variance and phenotypic variance ($T^{2}$) was also low (<0.10) and did not significantly differ between FR (Table 5). Thus, there is no evidence that the potential of the population to respond to selection depends on FR. However, the contribution of social genetic effects to the heritable variance was higher for animals on $R$ than on $F$. Thus, the ratio between $h^{2}$ and $T^{2}$ was around 0.52 for animals on $R$ and 0.86 for animals on $F$ (Fig. 2). Feeding regimen also affected the sign and magnitude of the correlation between direct and social genetic effects which was negative and moderate for animals on $R$ (posterior mean = −0.51; posterior sd = 0.22) and did not significantly differ from 0 for animals on $F$ (Table 5).

Figure 3 shows the correlations between direct ($d$) and social ($s$) genetic effects for animals on $F$ and $R$ which all differ significantly from 1. The correlation between direct genetic effects for animals on $R$ and direct and social genetic effects for animals on $F$ were both clearly positive, with posterior means of (posterior sd) 0.64 (0.14) and 0.56 (0.20), respectively. The correlation between direct genetic effects for animals on $F$ and social genetic effects for animals on $R$ was high and clearly negative (posterior mean = −0.47; posterior sd = 0.21), whereas the correlation between social genetic effects for animals on both feeding regimens did not significantly differ from 0.

### Relationship between models

The posterior mean (posterior sd) of the correlation between litter effects from Models 1 and 2 was very high, i.e. 0.94 (0.06). Regarding the genetic correlations, Table 6 shows the Pearson (upper diagonal) and Spearman (lower diagonal) correlations between the posterior means of individual EBV from Model 1, which ignores social genetic effects, and the posterior means of individual estimated total breeding values (ETBV) and its components from Model 2, which includes those effects. Pearson and Spearman correlations were very similar for all combinations of genetic effects. Therefore, all the comments provided below refer to Pearson correlations. Regarding the interaction between genotype and FR, when social genetic effects were ignored, the correlation between direct genetic effects (i.e. EBV) for animals on $F$ and $R$ was high and positive (0.94), which indicated that there was no G × FR interaction apart from a scale effect that originated from the effect of FR on the genetic variance. On the contrary, when social genetic effects were taken into account, the correlation between ETBV for animals on $F$ and $R$ was null (posterior mean = 0.23; posterior sd = 0.26), which indicated that a strong interaction between genotype and FR exists. This result originates from the structure of the correlations between direct and social genetic effects on FR as described above.

**Table 6:** Pearson (upper diagonal) and Spearman (lower diagonal) correlations between estimated breeding value (${\text{EBV}}$) from Model 1 and estimated total breeding value (${\text{ETBV}}$), direct genetic effects ($d$) and social genetic effects ($s$) from Model 2 for average daily gain of growing rabbits under full ($F$) or restricted ($R$) feeding regimen

	EBV_F	EBV_R	ETBV_F	ETBV_R	d_F	d_R	s_F	s_R
EBV_F		0.943	0.98	−0.047	0.985	0.834	0.119	−0.636
EBV_R	0.938		0.972	0.105	0.905	0.93	0.391	−0.602
ETBV_F	0.977	0.969		0.007	0.967	0.918	0.266	−0.66
ETBV_R	−0.028	0.101	0.013		−0.195	−0.014	0.761	0.689
d_F	0.983	0.898	0.964	−0.168		0.829	0.01	−0.733
d_R	0.817	0.917	0.909	−0.035	0.819		0.463	−0.734
s_F	0.115	0.362	0.252	0.741	0.018	0.424		0.181
s_R	−0.595	−0.572	−0.628	0.675	−0.696	−0.726	0.172	

The correlation between EBV and ETBV for animals on $F$ was very high and positive (0.98). This was due to the high and positive (0.99) correlation between direct genetic effects from Models 1 and 2, the low and positive (0.12) correlation between EBV and social effects from Model 2, and the small contribution of social effects to the total heritable variance under these conditions. However, for animals on $R$, the correlation between EBV and ETBV was almost negligible (0.11) as a result of a high and positive correlation between EBV and direct genetic effects from Model 2 (0.93), which is counteracted by a negative and moderate to high (−0.60) correlation between EBV and social genetic effects from Model 2. This is indicative of a sizeable contribution of social genetic effects to ETBV.

The correlation between EBV for animals on $F$ and ETBV for animals on $R$ was negative and very small (−0.05), which was mainly due to the negative and moderate to high (−0.64) correlation between EBV for animals on $F$ and social genetic effects for animals on $R$ (which account for 48% of the total heritable variance), since the correlation between direct genetic effects for animals on $F$ and $R$ was high and positive (0.83).

On the contrary, the correlation between EBV for animals on $R$ and ETBV for animals on $F$ was high and positive (0.97). This was the result of the high and positive correlation between EBV for animals on $R$ and direct genetic effects for animals on $F$ (0.91), the low to moderate and positive correlation (0.39) between EBV for animals on $R$ and social genetic effects for animals on $F$, and the small contribution of social effects to total heritable variance for animals on $F$.

## Discussion

Our findings indicate that although the ratio between total heritable variance and phenotypic variance did not differ significantly between FR, there was a higher contribution of social interaction effects to the heritable variance of animals on $R$ than on $F$. Feeding regimen also affected the sign and magnitude of the correlation between direct and social effects, which was negative and moderate for animals on $R$ and not significantly different from 0 for animals on $F$. In addition, the correlation between ETBV for animals under both FR was null, which indicates very strong G × FR interactions. This correlation results from the correlations between direct and social effects for animals on $F$ and $R$ that ranged from −0.47 (direct effects for animals on $F$ and social effects for animals on $R$) to 0.64. Therefore, selection of rabbits for ${\text{ADG}}$ under $F$ may be completely ineffective to improve ${\text{ADG}}$ in rabbits raised on $R$ when animals are housed in groups.

Feed restriction has been demonstrated to have good effects on the health and productive performance of growing rabbits (see review by Gidenne et al. [5]). Although weight gain is reduced during the period of feed restriction, a limitation of the amount of food reduces the risk of digestive diseases and improves feed efficiency both during restriction and especially after it, when the amount of food is gradually increased. As a consequence, feeding costs and the use of antibiotics are reduced, which result in economic profit and a reduced environmental impact of rabbit meat production. Thus, feed restriction has become a common management technique on commercial farms in Europe.

In our study, feed restriction was performed by supplying 75% of the ad libitum intake of a pelleted feed, once per day (~8 a.m.) in a feeder with three places, to breed rabbits that were housed in cages of eight individuals of similar size at weaning. These are standard conditions for rabbit meat production. According to Gidenne et al. [5], this technique of feed distribution is appropriate to achieve a good control of post-weaning intake and health status of the animals. This was confirmed by the 5.1% lower mortality rate for kits on $R$ than on $F$ that we observed in our study, which means an improvement of 35% over $F$ conditions. This result is in agreement with other findings in rabbits by Boisot et al. [22], Gidenne et al. [3] or Romero et al. [23].

Feed restriction modifies the feeding behaviour of growing rabbits who adapt to it very quickly. Rabbits on $F$ eat 30 to 40 meals throughout the day [24]. Conversely, rabbits on $R$ eat ~40% of the ad libitum intake within 2 h after feed distribution and complete their total intake within 10 h [25]. In our experiment, we also observed this feeding behaviour, which led to a high competition for food at distribution time with clear signs of antagonistic behaviour [4]. This could be a matter of concern for animal well-being. In spite of this feeding behaviour, Tudela and Lebas [26] pointed out that within-group variability of individual weight was not affected by feed limitation in collective cages because of the limited volume of the rabbit’s stomach, which limits the amount of feed an animal can eat in one meal. This means that all kits have access to food at some time of the day and therefore, roughly the same level of feed restriction is applied to all cage mates. However, in our experiment, we observed significant differences between FR in the within-group homogeneity of ${\text{ADG}}$ during the first 2 weeks and in the within-group homogeneity of ${\text{BW}}$ during the last 2 weeks. Some animals grew at different rates and this could be related to differences in the amount of feed ingested, which could be caused or not by competition for feed, or to differences in feed efficiency, which could be more visible when the amount of feed is limited. Therefore, it was necessary to explore the role that social interactions between cage mates may have on the genetic determinism of growth and feed efficiency when the amount of food is limited. It was also important to explore the consequences of selection for growth and feed efficiency under specific conditions of feeding regimen and housing on the productive performance of young rabbits bred under different conditions. For example, it was necessary to evaluate the productive performance of growing rabbits on $R$ that are housed in collective cages on commercial farms when breeding animals come from a nucleus herd in which selection for ${\text{ADG}}$ is performed on animals bred on $F$ in collective cages or on $R$ in individual cages.

In order to achieve this objective, ${\text{ADG}}$ of animals under different FR were assumed to be different traits following a character state model [6] for the analysis of G × E interactions. Two models were fitted to each trait: a classical repeatability animal model and an extension of that model including social genetic effects between cage mates. It was not possible to better consider the covariance structure of the longitudinal data by fitting a random regression model because of convergence problems of the sampling procedure. Since the animals were mixed, this model would have allowed the assessment of how social genetic and environmental effects change over time in growing rabbits. In fact, in a study on pigs, Canario et al. [27] and Camerlink et al. [28] highlighted that the association between social genetic effects and agonistic interactions, which is observed after mixing animals, decreased after several weeks. However, according to Piles et al. [29], the repeatability model could be a proper approximation for selection if there is no need to change the pattern of growth over time.

The models used in this study assumed that the residual variance was homogeneous with time and with body weight at weaning. In a preliminary analysis (results not shown), we demonstrated that residual variance was homogeneous with body weight at weaning but not with week (DIC heteroscedastic Model 1 = 231,366). However, since the estimates of other variance components of the models did not differ between the homoscedastic and heteroscedastic models, we present only the results of the homoscedastic models for the sake of simplicity.

The results from the classical repeatability model indicated that heritability of ${\text{ADG}}_{F}$ was low (0.08) compared to published estimates of this parameter in the same (0.15; [30]) or other rabbit populations (0.11 [31]; 0.18 [32] and [33]; 0.29 [34]). However, it is important to note that the definition of the trait and the model used for analysis were different in our study. In the previously published studies, ${\text{ADG}}$ was computed as the difference between body weight at the beginning and end of the fattening period divided by the number of days elapsed (ranging from 28 to 42 days), whereas we analysed weekly measures of ${\text{ADG}}$. It is well known that heritability increases with the length of the period measured because the residual variance is reduced by averaging the observations over a longer time period [35]. Therefore, estimates from those studies are not directly comparable with ours. The limited control of the environmental conditions on the experimental farm might have contributed to the large residual variance that we found.

The effect of the interaction between genotype and FR on ${\text{ADG}}$ has been documented in several species such as mouse [36–38], pig [39], mink [40] and rabbit [2] by comparing, in most of the studies, the performance of a small number of individuals raised under different feeding regimens. However, the magnitude of the variance of this interaction effect and its components was not previously estimated in rabbit.

In rabbits, Drouilhet et al. [2] compared BW at 63 days of age, ${\text{ADG}}$, ${\text{FI}}$, feed conversion ratio and ${\text{RFI}}$ of growing rabbits from two lines that were selected for feed efficiency following different strategies (selection for ${\text{ADG}}_{R}$ and selection for ${\text{RFI}}$) and bred on $F$ and $R$. The authors observed a significant interaction between FR and line effects only for ${\text{ADG}}$ but not for the other traits. Both lines had similar ${\text{ADG}}$ when raised on $R$ but the line selected for ${\text{ADG}}_{R}$ had a higher ${\text{ADG}}$ when raised on $F$ than that selected for ${\text{RFI}}$ (48.51 ± 0.72 vs. 45.29 ± 0.68 g/day, respectively). In addition, FR affected the phenotypic variance of the traits in a different way in the two selected lines depending on the trait. ${\text{ADG}}$ was less variable in the line selected for ${\text{ADG}}_{R}$ than in the line selected for ${\text{RFI}}$ when raised on $R$ (sd = 1.95 vs. 2.76 g/day, respectively) but was more variable in the line selected for ${\text{ADG}}_{R}$ than in the line selected for ${\text{RFI}}$ when raised on $F$ (sd = 4.48 vs 3.55 g/day, respectively). However, contrary to our results, ${\text{ADG}}_{R}$ was moderately to highly heritable (0.22 ± 0.06), which suggests that the animals were able to express their genetic potential for growth even when the amount of food was limited. However, unlike in our experiment, animals were housed in individual cages and therefore, competition for feed did not occur. Taken together, the different results on the genetic determinism of ${\text{ADG}}_{R}$ that were obtained between the experiment performed by Drouilhet et al. [2] and our experiment and the results that are reported by Dalmau et al. [4] on the differences in kit behaviour under different FR indicate that it is likely that social interactions played an important role in our experiment.

The social effect of an individual refers to its effect on the trait value of a social partner. It can be of environmental or genetic origin and generates an additional level of heritable variation, which is not part of the observed phenotypic variance of the individual trait. As a result, the heritable variance of socially affected traits can exceed the phenotypic variance and, thus, ignoring these effects could lead to the absence of an optimal response or even to a response in the opposite direction to that of the selection objective [17, 18].

In our experiment, the proportion of variance between groups relative to the phenotypic variance was equivalent under both feeding regimens and small (around 2% of the phenotypic variance). In spite of this, the environmental component of social effects may still be large if the covariance between direct and social environmental effects is strongly negative, as may occur for animals on $R$.

The ratio between total heritable variance and phenotypic variance was the same irrespective of the FR. However, the contribution of the social genetic effects to the heritable variance was higher for animals on $R$ than on $F$. The estimated values indicated that almost 50% of the heritable variance would be hidden when the classical model is used for selection to increase ${\text{ADG}}$ in growing rabbits on $R$, but only 14% when they are on $F$. The strategy used to reduce the initial variation in body weight may have reduced competition between cage mates. However, the effect of maternal and pre-weaning environmental effects on the estimated direct and social interaction effects for ${\text{ADG}}$ at fattening is expected to be minimized. In pigs, Bergsma et al. [19] estimated direct and social genetic effects for ${\text{ADG}}$ during fattening by distinguishing between a sub-population of animals fed ad libitum and the ~90% of animals that were fed on a restricted regimen. In their case, the residual variance for ${\text{ADG}}$ differed significantly between FR and was larger under $R$. Although the FR were less contrasted in their study than in ours, estimates for $T^{2}$ did not differ between FR.

Interestingly, in our study on rabbit, the sign and magnitude of the correlation between direct and social genetic effects differed between the two FR. It was negative and moderate for ${\text{ADG}}_{R}$, whereas it did not statistically differ from 0 for ${\text{ADG}}_{F}$. This means that, if social genetic effects are ignored, selection for increased ${\text{ADG}}_{R}$ could lead to more competitive animals, which would have a negative effect on the growth of their cage mates, whereas when feed is not limited this is not expected to happen. Conversely, Bergsma et al. [19] obtained similar non-significant correlations between direct and social genetic effects in pigs under two FR.

In addition, the consequences of ignoring social effects on selection decisions to increase ${\text{ADG}}$ in growing rabbits would be minimal when they are raised on $F$ (the rank correlation between EBV from Model 1 and ETBV from Model 2 for animals on $F$ was equal to 0.98) but they could be very important when they are raised on $R$ given the absence of correlation between estimated genetic values of a selection candidate from both models (the rank correlation between EBV and ETBV for animals on $R$ was equal to 0.11). Therefore, response to selection could be impacted if social effects are not taken into account in a breeding program for increasing ${\text{ADG}}_{R}$ when animals are kept in collective cages. This result stresses the importance of considering social genetic effects in selection programs, as a potential method to improve production and eventually reduce harmful behaviours in livestock [18, 27].

In a study on Japanese quail that were raised under feed restriction and housed in 16-bird cages, Muir [16] found a moderate to large and negative (−0.56) genetic correlation between direct and social effects on weight at 6-weeks, which is comparable to that obtained in our experiment. In pigs fed ad libitum in groups of 6 to 12 pen mates of the same sex, Bergsma et al. [19] found a positive but low value for this parameter, which also agrees with our results. However, Chen et al. [41] reported positive to negative social interactions for growth in pig populations from the United States housed in groups of 15 pen-mates, with a maximum negative value at −0.37. Canario et al. [42] found that direct and social effects were independent in pigs raised under feed restriction. In mice and pigs, social genetic effects are known to favour antagonistic behaviours when mixing animals [27, 43].

The magnitude of the interaction between genotype and feeding regimen is mainly due to the covariance structure of the direct and social genetic effects under different FR, which results in a null correlation between total heritable effects for animals on $R$ and $F$. All the correlations between combinations of direct and social genetic effects for animals on $F$ and $R$ differed significantly from 1. The most remarkable result may be the correlation between direct genetic effects for animals on $F$ and social genetic effects for animals on $R$, which was high and clearly negative. This result suggests that animals with high genetic potential for growth on $F$ could be those that display a more competitive behaviour when they are feed restricted. Thus, the environment modulates the expression of social skills. Competition for food favours animals that have a less altruistic attitude towards cage mates. As a consequence, the productive performance of growing rabbits raised under feed restriction and housed in collective cages may differ greatly from that expected when selection for ${\text{ADG}}_{F}$ is performed in a nucleus herd.

The consequences of ignoring social genetic effects would be minimal if animals were selected on $R$ and produced on $F$ (rank correlation between EBV for animals on $R$ and ETBV for animals on $F$ was 0.97) because of the low contribution of social genetic effects to ${\text{ADG}}_{F}$. However, if animals were selected on $F$ and produced on $R$, no genetic response would be achieved on production farms because of the null rank correlation between EBV for animals on $F$ and ETBV for animals on $R$ (−0.028).

To date, few selection experiments that include social genetic effects have been performed in livestock species. In Japanese quail [16], two lines were selected for increased weight at 6 weeks under feed restriction (i.e. feeding was limited to once per day and access to the feeder was restricted). One of the lines was selected only for direct effects while the other was selected for ETBV using an index. After 23 cycles of selection (i.e. six generations), selection based on ETBV led to a positive response of 0.52 ± 0.25 g/hatch, whereas selection based only on direct genetic effects led to no response on weight at 6 weeks and to an increase in mortality of 0.32 ± 0.15 deaths/hatch. Therefore, ignoring social genetic effects was detrimental not only to response to selection but also to animal well-being. Selection for direct genetic effects only worsened the social genetic effects, which explains the lack of response in this line because responses in direct and social effects were in opposite directions.

These results were confirmed in a later experiment that was carried out on the same population under the same management and environmental conditions [44]. In this case, multilevel selections with birds housed in either kin or random groups were compared. Selection was based on best linear unbiased predictions (BLUP) of breeding values (EBV) which, when relatives are in the same group, is equivalent to multilevel selection since BLUP also weights the group performance. On the contrary, if relatives are in different groups, EBV do not include any weight in group performance. Results over 18 selection cycles indicated that response with multilevel selection in kin groups was 1.30 g/hatch, which was greater than that obtained with multilevel selection in random groups (1.13 g/hatch) and also significantly greater than that with selection based on ${\text{TBV}}$ (0.52 ± 0.25 g/hatch). In addition, the mortality rate was also significantly lower (6 vs. 8% in kin and random groups, respectively). In our current analysis, the consequences on rabbit survival were not evaluated since it was observed that mortality was mainly due to some bouts of ERE on the farm. This is an infectious disease, which clearly impairs animal growth, but it is not considered to be caused by antagonistic behaviour between cage mates (animals in individual cages also have ERE). However, the impact that individuals have on each other is a crucial factor for the prevalence of infectious diseases in animals that are housed in groups [45, 46].

## Conclusions

Social genetic effects contribute largely to the total heritable variance of average daily gain when growing rabbits are raised under restricted feeding but not when they are fed ad libitum. Ignoring those effects in a breeding program for increasing rabbit growth is likely to have negative consequences on the productive performance of young rabbits and eventually on animal well-being when the amount of food is limited. The interaction between genotype and feeding regimen will lead to a substantial re-ranking of the selection candidates under different conditions because of the null correlation between total heritable variance for animals on $R$ and $F$. This is mainly due to the null and negative genetic correlations between direct and social genetic effects on full and restricted feeding regimen, respectively. Therefore, we recommend to select animals under the same conditions of feeding and housing as those applied on production farms for rabbit meat production, especially when feed restriction is applied on commercial farms.
